# Highly Sensitive Graphene/Polydimethylsiloxane Composite Films near the Threshold Concentration with Biaxial Stretching

**DOI:** 10.3390/polym12010071

**Published:** 2020-01-02

**Authors:** Anqi Liu, Zhengji Ni, Juan Chen, Yuanshen Huang

**Affiliations:** 1 School of Optical Electrical and Computer Engineering, University of Shanghai for Science and Technology, Shanghai 200093, China; an2428932469@163.com (A.L.); sioi@usst.edu.cn (Z.N.); cj0152866@163.com (J.C.); 2 Shanghai Key Laboratory of Modern Optical Systems, Engineering Research Center of Optical Instruments and Systems, Shanghai 200093, China; 3 Shanghai Institute of Optical Instruments, Shanghai 200093, China

**Keywords:** threshold concentration, biaxial tension, graphene, PDMS

## Abstract

Uniformly dispersed graphene effectively improves the strain-sensing capability of the composite film under a low graphene load in nanocomposites prepared with polydimethylsiloxane (PDMS) and graphene (GNP) monolayer powder. The threshold concentration of graphene was determined by loading nanocomposites at different temperatures. For different concentrations, when using traditional uniaxial stretching, the rate of resistance change of films near the threshold concentration is five times higher than the rate of films with a high concentration. Compared with traditional uniaxial stretching, the biaxial stretching we introduced can effectively improve the sensitivity of the film by an order of magnitude. The change in the resistance of the film near the threshold concentration is due to the change of the tunnel length and the cross-section of the tunnel, whereas the high concentration of the film is due to the change of the conductive path inside the film. Biaxial stretching has different effects on films with different concentrations, but the final effect of increasing sensitivity is the same. This study provides guidance for improving the strain-sensing sensitivity of GNP/PDMS composite films and the application of biaxial tension in detecting human motions.

## 1. Introduction

The strain sensor based on the change of resistance by mechanical deformation has attracted great attention because of its extensive application in health monitoring and motion detection [1]. Polymer-based strain sensors have a fast response in the form of resistance changes when subjected to tensile or compressive strains, while also meeting the requirements of high sensitivity, good repeatability, and wide test range for deformation monitoring [2,3,4,5,6,7]. This kind of strain sensor is usually fabricated by dispersing one or more electrically conductive fillers, such as carbon black (CB) [8,9,10], carbon nanotubes (CNTs) [11,12,13,14,15], and graphene (GNP) [16,17,18,19], in the insulating polymeric matrix. In addition to the above materials, metal coatings can also be chemically deposited on polymers as conductive materials. But this method is not as convenient as the doped composite films which can control the concentration [20]. In the selection of conductive fillers, Zheng et al. found that the sensitivity and repeatability of CNTs were superior to that of CB by comparing conductive composites with CB and CNTs as fillers [21]. However, at present, CNTs are prone to agglomeration during processing, and the number of high-quality nanotubes is limited and the production cost is high [22]. As nanotubes are based on building blocks of all graphite allotropes, graphene sheets offer an alternative to nanocomposites due to their excellent properties and the natural content of graphite [5].

In the application of graphene, some researchers, who studied graphene in combination with different substrates, found that the response of GNP/PDMS (polydimethylsiloxane) to electrical resistance was three or four orders of magnitude higher than that of GNP/Si, GNP/PET (polyethylene terephthalate) [23], and GNP/PU(polyurethane) [24]. The composite prepared by the combination of the elastic PDMS and the conductive graphene has been used to make stretch-sensitive strain sensors [25,26,27,28,29]. Dong et al. found that the deformation range and resistance change rate of 5 wt. % samples were better than that of 3 and 8 wt. % in the study of different concentrations of GNP–PDMS composites [30]. According to the conductivity curve of GNP/PDMS, the resistance change rate of films near the threshold concentration is more than one order of magnitude higher than that of films with a high concentration. Although 5 wt. % is better in high-concentration samples, there is still a lack of research near the threshold concentration. The application of a GNP/PDMS film is basically the application of uniaxial stretching [31], and there are few studies on other stretching methods.

In this article, we prepared a GNP/PDMS composite film near the threshold concentration by combining a single layer of graphene and polydimethylsiloxane. When the outside temperature changes, the concentration of the composite film changes, and the resistance resulting from temperature can be measured in real time. If the concentration changes to near the threshold concentration, there will be a significant change in resistance. In addition, compared with traditional uniaxial stretching, we can get higher gauge factor (GF) values and greater sensitivity with biaxial stretching. The bending motion of the knee can be detected by applying biaxial stretching to the knee’s stretch sensor.

## 2. Determination of Threshold Concentration

### 2.1. Theoretical Threshold Concentration

In terms of the classical seepage theory, the relationship between conductivity and packing concentration is δ = δ_0_(P − P_0_)^t^, where δ is conductivity, δ_0_ is scaling factor, P is the concentration of GNP, and P_0_ is the threshold concentration of GNP. When δ is 1.25 × 10^−6^, δ_0_ is 6.15 × 10^−4^, P is 2 wt. % and t is 1.3, the threshold concentration calculated theoretically is 0.98 wt. % (0.445 vol. %) [21].

### 2.2. Materials and Preparation

Single-layer graphene powder was purchased from Chinese Jiangsu XFNANO Co. LTD. (Jiangsu, China), with a thickness of 0–0.8 nm, a diameter of 0.5–5 μm, a density of about 2.25 g/cm^3^, and a single-layer ratio of 80%. We bought polydimethylsiloxane (Sylgard 184 Silicone Elastomer) and curing agent from Dow Corning (Midland, MI, USA).

**Figure 1:**
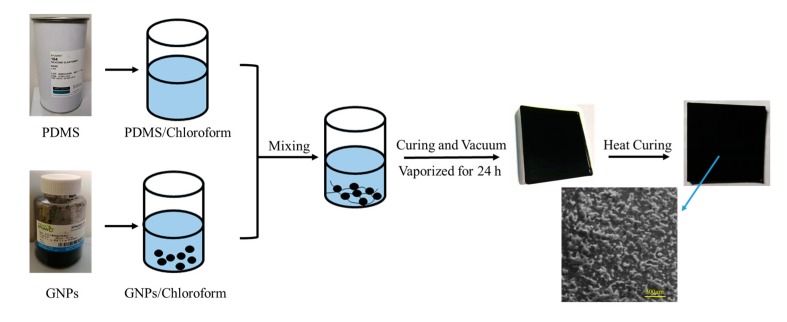
Process for the fabrication of graphene and polydimethylsiloxane (GNP/PDMS) nanocomposites.

We elaborated the fabrication process of GNP/PDMS nanocomposites in Figure 1. In this study, the nanofillers were uniformly dispersed in a high-viscosity PDMS base polymer using volatile chloroform (Samchun, Seoul, Korea). For the detailed method, the GNP powder was first added to chloroform, then stirred for 0.5 h to sufficiently disperse the nanofiller. PDMS was diluted with chloroform at a 1:1 ratio and stirred for 0.5 h to lower its viscosity, which promoted uniform dispersion of the filler. The diluted PDMS was added to the GNP suspension, and the mixture was stirred for an additional 0.5 h. A curing agent (PDMS to curing agent mass ratio of 10:1) was then dripped into the mixture and stirred for 0.5 h. The mixed solution was evacuated in a vacuum box and placed on a glass substrate at room temperature all day to completely evaporate chloroform so as not to affect the final properties of GPN/PDMS. Finally, we made the cured the mixture at 100 °C for 0.5 h in the oven. After obtaining the film, we observed the dispersion of graphene inside the film by means of electron microscope scanning, as shown in Figure 1. Graphene was uniformly dispersed in PDMS.

The film samples, each with an area of 6 cm × 6 cm and thickness of 1 mm, were prepared for the sensing test. The GNP/PDMS composite film was installed on a drawing device to stretch the GNP/PDMS composite film. Real-time resistance tests under mechanical strain and at different temperatures were performed on a high-voltage megohmmeter (SMART SENSOR, AR3127, Shenzhen, China) connected to a computer. The double-probe resistivity method was used to measure the volume resistance of the GNP/PDMS composite film under various mechanical strains and temperature changes.

### 2.3. Heating Experiments

To get the threshold concentration of the GNP/PDMS composite film, the GNP/PDMS composite film is set down on a heating stage that could change the temperature of the film. When the film is heated, the proper conductivity of GNP is almost unaffected, and the change of the characteristics of PDMS, such as permeability and solubility-absorption, have little effect on the conductivity of GNP. As for the influence of humidity on the conductivity of the film, the sensor can be used without humidity compensation if the humidity of the external environment is relatively stable [32]. As a result of the thermal expansion properties of PDMS [33], the volume of PDMS changes when the outside temperature changes, and the concentration of the film varies with the volume. The volume resistance was obtained using a two-probe resistivity method.

An experiment of the temperature change on 1 wt. % of the sample was performed to determine the actual threshold concentration of the GNP/PDMS nanocomposites. A range of different temperature conditions were applied to 1 wt. % GNP/PDMS nanocomposite samples. The temperature varied between 25 and 110 °C, increasing by 5 °C each time. For measuring the change in resistance at different temperatures, an unvarying voltage of 200 V was continuously acted on the filler during the resistance measurement. The relationship between the resistance change curve and temperature of 1 wt. % GNP/PDMS nanocomposites at different temperatures was recorded.

**Figure 2:**
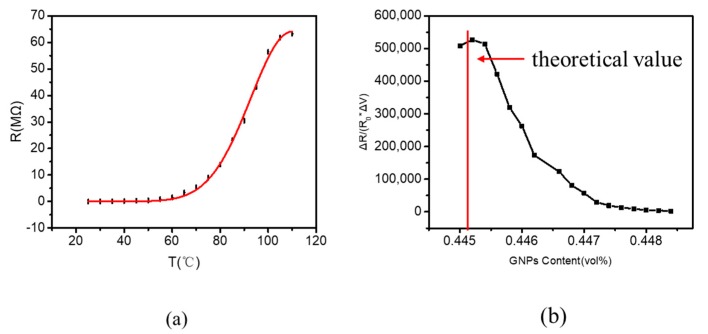
(**a**) Resistance of 1 wt. % GNP/PDMS films at different temperatures. (**b**) Curve between the resistance and the volume concentration of the films varies with temperature.

As shown in Figure 2a, the resistance of 1 wt. % GNP/PDMS nanocomposites increased significantly upon the increasing temperatures. When the temperature varied between 25 and 70 °C, the resistance change of the sample was relatively flat. When the temperature was ascended from 75 to 100 °C, the sample resistance increased sharply. At temperatures above 100 °C, the resistance of the sample tended to be flat again. The measured data points were basically consistent with the fitting curve of the formula. It can be concluded from Figure 2b that when the temperature reached 100 °C, the value of $∆R/\left(R_{0}\times ∆V\right)$ reached the maximum 527, 101, where ∆R represents the variation of resistance, $R_{0}$ represents the initial resistance, and ∆V is the change in volume with temperature. Therefore, it can be suggested that there is a threshold concentration of GNP/PDMS nanocomposites at a temperature of approximate 100 °C. Once the temperature changes to 100 °C, the volume concentration changes to 0.003 vol. %, and the mass concentration changes to 0.99 wt. %, which basically agrees with the theoretical value. If the temperature is higher than 100 °C, the sample concentration is lower than the threshold concentration and can be approximated seen as an insulator.

When the concentration of graphene approached the threshold of the composite, the conductivity of the composite was dominated by the tunneling effect. The thickness of the film, the concentration of the graphene, the distance to the tunnel, the size of the graphene, and so on, all affect the tunneling resistance. The tunneling resistance (R) can be expressed [34] as follows:

$$R=\frac{8\pi h\varphi d^{2}m}{Aq^{3}mV}exp\left(\frac{8\pi \sqrt{2m}d\varphi^{\frac{3}{2}}}{3hqV}\right),$$

where A is a cross-sectional area of tunnel, q is the electronic charge, m is free electron mass, V is voltage, h is Planck’s constant, d is separation between the two adjacent graphene, and φ is barrier height. When the temperature increases, the concentration of the film decreases and the length of the tunnel increases. Although the cross-sectional area of the tunnel has a negative effect on the tunneling resistance, the overall tunneling resistance of the thin film is still increasing. The actual measured data are in agreement with the trend of the fitted curve of the formula in Figure 2a.

## 3. Experiments

### 3.1. Uniaxial Stretching

When the concentration of graphene increases, the conductivity of the film is dominated by the conductive path inside the film, rather than the tunneling effect [35,36]. We prepared a 1.5 wt. % sample. The effects of these two different conductive mechanisms on the rate of change of film resistance can be compared by simple uniaxial stretching.

**Figure 3:**
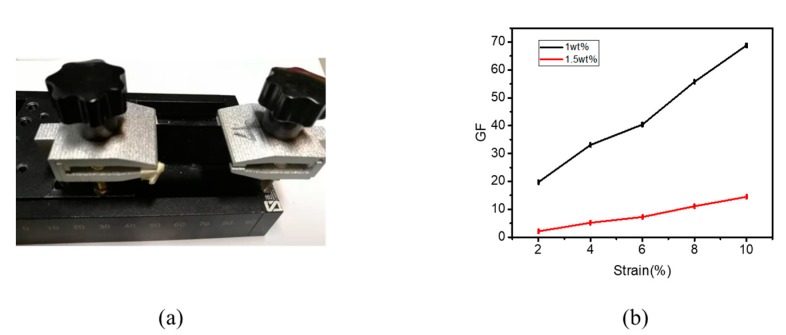
(**a**) Uniaxial stretching fixture. (**b**) GF (gauge factor) values of 1 wt. % film and 1.5 wt. % film during uniaxial stretching.

Uniaxial stretching with the same amount of tension was performed on both films near the threshold at 1 wt. % and at a higher concentration of 1.5 wt. %. To specify the strain sensitivity of the GNP/PDMS nanocomposite, we calculated the GF value (R − R_0_)/(R_0_ × ε), where R_0_ is the initial resistance of the strain sensor, R is the corresponding resistance when the tensile strain changes, and ε is the amount of stretching. From the GF values in Figure 3b, the GF value of 1 wt. % is five-times higher than that of 1.5 wt. %. Therefore, when the concentration of the film is near the threshold, the sensitivity of the film is better than that of the high concentration.

### 3.2. Comparison of Different Stretches

**Figure 4:**
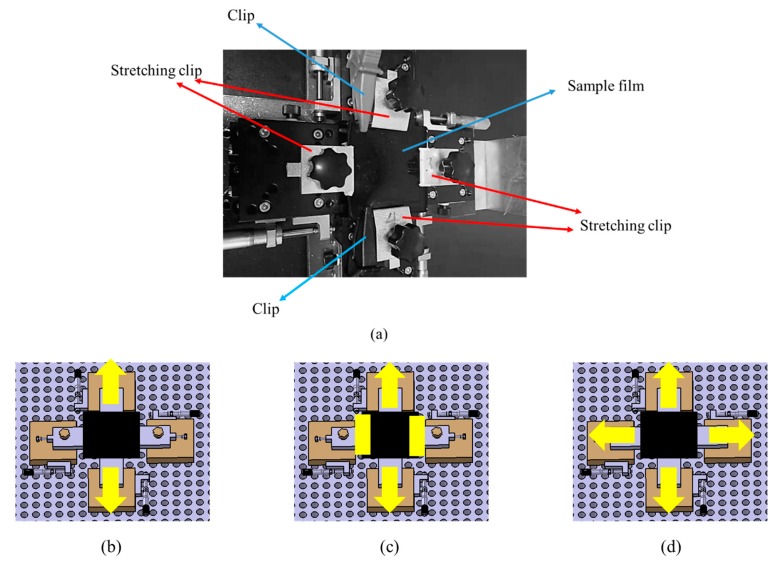
(**a**) Biaxial stretching fixture. (**b**) First stretching method. (**c**) Second stretching method. (**d**) Third stretching method.

From the formula of tunneling resistance, when the concentration of the film is fixed, the length of the tunnel and the cross-sectional area of the tunnel can be considered as the parameters that can change the tunneling resistance. We used three different stretching methods of the fixture, as shown in Figure 4a. The first is where there is only one direction of stretching, and the other direction is free. The second is to hold it in one direction with a clamp and stretch it in the other direction. The last one is to stretch in both directions at the same tensile strength. The three diagrams are shown in Figure 4b–d.

### 3.3. Results and Discussion

**Figure 5:**
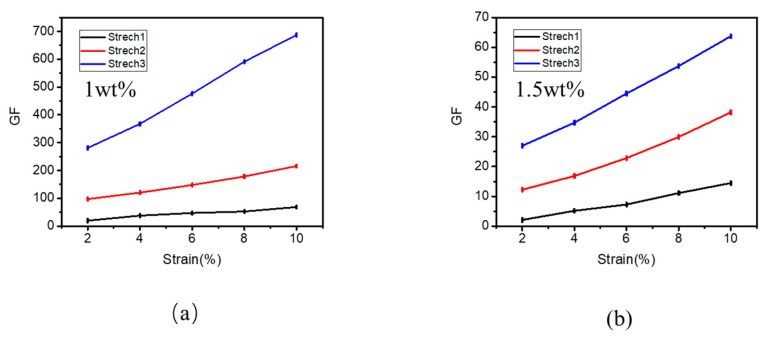
(**a**) GF values of GNP films with a concentration of 1 wt. % under different stretching modes. (**b**) GF values of GNP films with a concentration of 1.5 wt. % under different stretching modes.

In Figure 5a, the influence of three different stretching modes on the sensitivity of films can be seen. The GF value of 1 wt. % films increased from 19.69 to 68.81 when stretched in the first way. The GF value of 1 wt. % films increased from 158.31 to 280.99 when stretched in the second way. The GF value of the film can be increased from 281.21 to 687.43 under the third stretching condition. In conventional uniaxial stretching, the length of the tunnel increases, but the cross-sectional area of the tunnel does not change much. The tunneling resistance is mainly affected by the tunnel length and cross-sectional area. Therefore, biaxial stretching is introduced to increase the length of the tunnel, while reducing the cross-sectional area of the tunnel to improve the rate of changing resistance of the film. Then, the GF value is affected by the rate of changing resistance. GF represents the sensitivity of the film. It can be seen that, compared with traditional uniaxial stretching, the introduced biaxial stretching can improve the sensitivity of the film by one order of magnitude.

At the same time, we also performed three kinds of stretching on the film of 1.5 wt. %. As Figure 5b shows, the GF value of the film changed from 2.08 to 14.46 during uniaxial stretching. The GF value of the second stretch state increased from 12.24 to 38.18. In the third stretch condition, the GF value of the film increased from 26.97 to 63.75. In the case of biaxial stretch, the GF value of 1.5 wt. % film did not reach an order of magnitude higher than the GF value of uniaxial stretch, but it was also five times higher than that of uniaxial stretch.

Biaxial stretch increases the sensitivity of films near the threshold concentration by increasing the tunnel distance and reducing the cross-sectional area of the tunnel, whereas biaxial stretch increases the sensitivity of films with high concentration due to the fact that it increases the damage degree of the conductive pathway inside the films. Although the mechanism of biaxial stretching on films with different concentrations is different, the final effect of increasing sensitivity is the same. Therefore, biaxial stretch is applicable not only to films near the threshold concentration but also to films with high concentration. The GF values of films near the threshold concentration is 10 times higher than GF values of films with a high concentration in the comparison of different concentrations. Thus, the highest sensitivity can be achieved when the film is biaxially stretched near the threshold concentration.

## 4. Application

**Figure 6:**
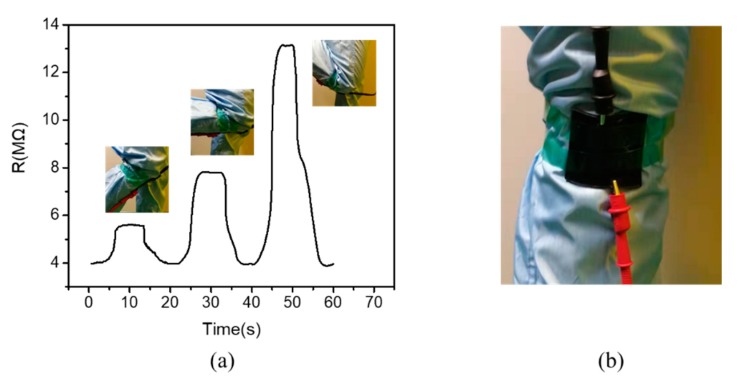
(**a**) Corresponding effect of the film on the knee joint bending at 45°, 90°, and 135°. (**b**) Schematic diagram of the film clamped on the knee.

As the concentration of the film is controlled near the threshold and biaxial stretching can improve the sensitivity, we can apply the stretching of the film to the response to human movements. The motion of the knee is a three-dimensional motion. When the knee joint bends, there will be three directions of x/y/z axes [37]. Thus, we fix the film on the knee joint. When the knee bends, the membrane undergoes biaxial stretching. The film of 1 wt. % was fixed on the knee joint, and the resistance of the film was measured when the knee joint was bent at different angles. Figure 6 shows the changes in the resistance of the membrane when the knee was bent at 45°, 90°, and 135°. Due to strain relaxation of PDMS, the response time of the membrane was 3–4 s when the knee was bent and recovered [38]. And our response time was 3 s. After the knee was bent, the resistance fluctuated in the range of 0.1 MΩ. When the knee returned to its original state, the resistance of the membrane returned to its original value. This shows that the film is stable and repeatable, and the response time is fast.

## 5. Conclusions

The threshold concentration of the sample film was determined to be 0.98 wt. % by using the thermal expansion property of PDMS. When the concentration of the composite film is controlled near the threshold, the electrical resistance of the film is mainly affected by the tunneling effect. But the resistance of high-concentration film is decided by the internal conduction path. Therefore, we compared samples near the threshold concentration of 1 wt. % with a higher concentration of 1.5 wt. % by traditional uniaxial tension. The sensitivity of the 1 wt. % sample is five times higher than that of the 1.5 wt. % sample. At two different concentrations, the biaxial tension which we refer to can improve the sensitivity ten times more than traditional uniaxial tension. Therefore, compared with conventional uniaxial stretching applications in human motion detection, biaxial stretching near the threshold concentration can improve sensitivity by an order of magnitude. In subsequent studies, biaxial stretching can also be applied to other joint flexions, such as the elbow. The response time of the film is also a problem to be considered later.
